# Establishment of age-specific reference intervals for peripheral blood SII, NLR, PLR, and LMR in healthy children

**DOI:** 10.1186/s12865-025-00797-2

**Published:** 2026-01-19

**Authors:** Xiaodan Zhang, Yaohui Song, Minggang Yin, Liangjun Zhang

**Affiliations:** 1 Department of Laboratory Medicine, Zigong First People’s Hospital, Zigong, Sichuan Province China; 2 Department of Laboratory Medicine, Zigong Fourth People’s Hospital, Zigong, Sichuan Province China

**Keywords:** Systemic immune-inflammation index, Neutrophil-to-lymphocyte ratio, Platelet-to-lymphocyte ratio, Lymphocyte-to-monocyte ratio, Reference intervals

## Abstract

**Objective:**

To establish and validate age-specific reference intervals for the peripheral blood systemic immune-inflammation index (SII), neutrophil-to-lymphocyte ratio (NLR), platelet-to-lymphocyte ratio (PLR), and lymphocyte-to-monocyte ratio (LMR) in healthy children.

**Methods:**

A total of 2106 healthy children undergoing physical examination at The First People’s Hospital of Zigong from April 2023 to February 2025 were enrolled. Blood cell parameters were measured using the XN-1000 hematology analyzer, and SII, NLR, PLR, and LMR were calculated. The non-parametric method was employed to establish the 95% reference intervals.

**Results:**

No statistically significant differences were observed in SII, NLR, PLR, or LMR between genders (*P* > 0.05). Participants were stratified into three age groups: 28 days to 2 years, 2 to 6 years, and 6 to 18 years. The established reference intervals for SII were 28.60 × 10⁹/L to 298.85 × 10⁹/L, 83.09 × 10⁹/L to 601.33 × 10⁹/L, and 140.02 × 10⁹/L to 657.19 × 10⁹/L, respectively, for the three age groups. The corresponding NLR reference intervals were 0.10–0.92, 0.35–1.88, and 0.57–2.30. PLR reference intervals were 29.39-115.15, 39.93-150.46, and 56.74-172.55. LMR reference intervals were 3.42–13.84, 2.81–13.03, and 2.41–10.56. A validation study conducted on 92 children from the Department of Child Health Care of the same hospital between April 2025 and September 2025 confirmed the applicability of these reference intervals. This indicates that the established reference intervals for peripheral blood SII, NLR, PLR, and LMR in children from the Zigong region are suitable for local clinical practice.

**Conclusion:**

This study is the first to establish reference intervals for NLR, PLR, SII, and LMR in children from the Zigong region, providing a basis for the assessment of inflammatory diseases in local pediatric populations.

## Introduction

In recent years, inflammatory biomarkers derived from routine complete blood count—specifically, the systemic immune-inflammation index (SII), neutrophil-to-lymphocyte ratio (NLR), platelet-to-lymphocyte ratio (PLR), and lymphocyte-to-monocyte ratio (LMR)—have gained prominence for their diagnostic and prognostic value across a spectrum of diseases. Their clinical utility is well-documented globally, including in studies involving diverse populations such as those in Mexico and India [1]– [2], covering conditions from infections and Kawasaki disease to cancers and autoimmune disorders [1–7].

Although reference intervals for the aforementioned indices have been reported and are applied in adult and pregnant populations [8–10], a significant gap persists due to the scarcity of systematically established pediatric reference intervals in China. This is critically important because the developing hematopoietic and immune systems in children produce hematological profiles that are fundamentally distinct from those of adults [11]. The direct application of adult reference intervals to pediatric patients risks misinterpreting normal developmental hematological patterns, potentially leading to diagnostic errors. This is because age-specific reference ranges for blood cell counts are fundamentally different in children [12]. For example, using adult thresholds could lead to the over-diagnosis of neutropenia in infants or young children whose normal neutrophil counts are lower, or underestimate the significance of lymphocytosis which is more common and often pronounced in pediatric viral infections [12].Although region-specific studies in China (e.g., Guangdong [13], Shenyang [14], Shenzhen [15]) have utilized ROC curve analysis to demonstrate the diagnostic potential of these indices in pediatric diseases, studies aimed at establishing reference intervals for healthy children - a necessary foundation for such clinical applications - are scarce and lack standardization.

To address this critical gap for children from infancy through adolescence, this study was designed to establish age-specific reference intervals for SII, NLR, PLR, and LMR in a rigorously defined cohort of healthy children aged 28 days to 18 years from the Zigong region of China.

## Patients and methods

### Patients

From April 1, 2023, to February 28, 2025, we consecutively recruited children who presented for routine health checkups at the Department of Child Health Care. A total of 2,234 children were initially enrolled. The sex distribution within the cohort reflected the natural attendance pattern and was not actively controlled. The inclusion criterion was defined as apparently healthy children aged 0–18 years from the Zigong region.

From April 1, 2023, to February 28, 2025, we consecutively recruited children presenting for routine health checkups at the Department of Child Health Care. Initially, 2,234 children were recruited. Written informed consent was obtained from the parents or legal guardians of all participating children.The sex distribution in the cohort was not actively controlled but reflects the natural attendance pattern under the consecutive recruitment scheme.This study was approved by the Ethics Committee of The First People’s Hospital of Zigong (Approval No. 03202024) and was conducted in accordance with the principles of the Declaration of Helsinki. The inclusion criterion was defined as healthy children aged 0–18 years from the Zigong region. Exclusion criteria were established in accordance with the guidelines outlined in “WS/T 779–2021 Reference Intervals for Child Blood Cell Analysis” [12], and included: (1) Diagnosis of congenital diseases; (2) Presence of fever or any acute diseases within the preceding two weeks; (3) History of chronic diseases, including hematological disorders (e.g., anemia, leukemia, platelet disorders), allergic conditions (e.g., eczema, urticaria, bronchial asthma), respiratory diseases (e.g., acute respiratory infections, lung malformations), urinary system diseases (e.g., Henoch-Schönlein purpura nephritis, glomerulonephritis, nephrotic syndrome), digestive system diseases (e.g., chronic diarrhea, inflammatory bowel disease), rheumatic immune diseases (e.g., rheumatoid arthritis, systemic lupus erythematosus), circulatory system diseases (e.g., myocarditis), endocrine and metabolic disorders (e.g., diabetes, thyroid diseases), malignancies, history of radiotherapy or chemotherapy, burns, or muscle trauma; (4) Underweight status, defined as a BMI-Z score less than − 3 for children aged 5 years and below according to WHO growth standards, or a BMI below the age-specific screening threshold for underweight according to the Chinese industry standard WS/T 456 for children aged 6–18 years; (5) Recent medical history including: medication use (therapeutic drugs or supplements such as antibiotics, corticosteroids, or vitamin C) within one week; or history of surgery, blood transfusion, or significant blood loss within one month; (6) Abnormal laboratory findings: white blood cell count < 3.0 × 10⁹/L or > 15.0 × 10⁹/L, hemoglobin < 90 g/L, or mean corpuscular volume (MCV) < 75 fL. The assessment of exclusion criteria was based on a combination of: (1) a standardized health questionnaire completed by the child’s parent or guardian, which covered medical history (including congenital diseases, chronic illnesses, recent acute illnesses, medication use, surgery, etc.), and (2) the results of the current health examination, including the physical examination findings and the complete blood count (CBC) parameters obtained from the Sysmex XN-1000 analyzer. Children were excluded if any positive indication was reported in the questionnaire or if their CBC parameters fell outside the predefined healthy ranges specified in the criteria.After applying the exclusion criteria, 2106 children were included in the final analysis.

### Methods

Before blood collection, all participants followed the standard pre-examination guidelines for routine health checkups, which included fasting for 8 to 12 h. Approximately 2 mL of peripheral venous blood was drawn into EDTA-K2 anticoagulant vacuum tubes and thoroughly mixed. Samples were stored at room temperature (18–25 °C) and analyzed within 1 h of collection to ensure the stability of cellular parameters, in accordance with international recommendations for hematological testing [16]. Blood cell parameters, including platelet, monocyte, lymphocyte, and neutrophil counts, were determined using the Sysmex XN-1000 hematology analyzer (Sysmex Corporation, Japan). Reagents, quality control materials, and calibration standards were exclusively Sysmex original products, stored and used strictly according to the manufacturer’s recommended conditions. Internal quality control was performed following the Westgard multi-rule system, applying the 1–3 S, 2–2 S, and R-4 S rules. External quality assessment was carried out by the Clinical Laboratory Center of the National Health Commission of China, and all results complied with the required standards.The SII, NLR, PLR, and LMR values were calculated using the following formulas: SII = (Platelet Count × Neutrophil Count) / Lymphocyte Count; NLR = Neutrophil Count / Lymphocyte Count; PLR = Platelet Count / Lymphocyte Count; LMR = Lymphocyte Count / Monocyte Count.

### Statistical analysis

**Fig. 1:**
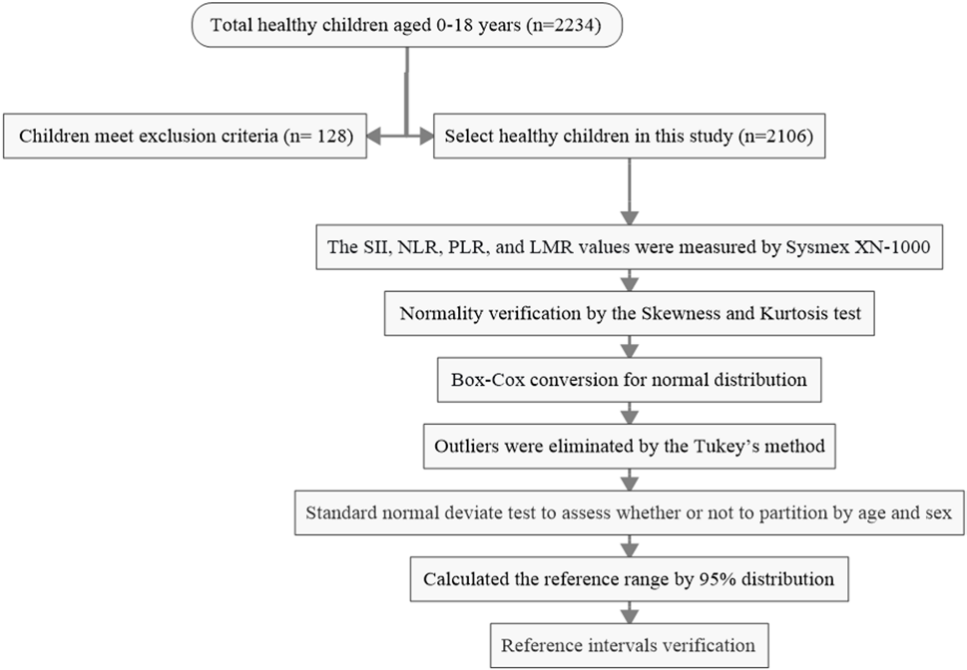
The flow chart of this study for establishing reference values

Statistical analysis was performed using SPSS software (version 27.0). Extreme outliers were excluded based on predefined criteria: white blood cell count < 3.0 × 10⁹/L or > 15.0 × 10⁹/L, hemoglobin < 90 g/L, or mean corpuscular volume (MCV) < 75 fL. Further outlier identification was conducted using Tukey’s method, where the lower bound was defined as P₂₅ − 1.5 × IQR and the upper bound as P₇₅ + 1.5 × IQR; values outside this range were considered outliers and removed. The normality of data distribution was assessed using the Kolmogorov-Smirnov test. Normally distributed quantitative data are expressed as mean ± standard deviation (x̅ ± S), while non-normally distributed data are presented as median (P₂₅, P₇₅). In accordance with the CLSI EP28-A3C guidelines [17], non-normally distributed data were subjected to Box-Cox transformation to approximate a normal distribution. For group comparisons, one-way ANOVA was used for comparisons across multiple groups, and the Mann-Whitney U test was applied for comparisons between two groups. A P-value < 0.05 was considered statistically significant. When a statistically significant difference was observed between groups, the Z-value and critical Z-value were compared to determine whether partitioning of reference intervals by sex and age was necessary. This partitioning decision was made following the recommendations of the CLSI EP28-A3c guideline [17]. The Z-value was calculated as Z* = 3 × √[(n₁ + n₂) / 240]. If Z > Z*, the between-group difference was considered clinically significant, warranting the establishment of separate reference intervals for the respective subgroups. To establish reference intervals, the nonparametric percentile method (P₂.₅ to P₉₇.₅) was employed, following the CLSI EP28-A3C guidelines [17]. The flow chart of this study is shown in Fig. 1.

## Results

### Distribution of data and elimination of outliers

This study followed the guidelines outlined in “WS/T 779–2021 Reference Intervals for Child Blood Cell Analysis” ^12^ issued by the National Health Commission of the People’s Republic of China. Participants were stratified into six age subgroups: 28 days to 6 months, 6 months to 1 year, 1–2 years, 2–6 years, 6–13 years, and 13–18 years. In accordance with the CLSI EP28-A3C guideline [17], reference intervals were established using a non-parametric approach. For skewed data distributions, the reference intervals were defined by the 2.5th to 97.5th percentiles [18]. The reference intervals for peripheral blood SII, NLR, PLR, and LMR were subsequently established and validated using this methodology. A 92 outliers were identified and removed using Tukey’s method, resulting in a final cohort of 2014 children (1015 males and 999 females). (Table 1)

**Table 1:** Hematological parameters of healthy children (*n* = 2014)

Age group	*n*	male	female	Platelet (×10⁹/L)	Neutrophil (×10⁹/L)	Lymphocyte (×10⁹/L)	Monocyte (×10⁹/L)
28 days-6 months	154	76	78	358(312,409)	1.41(1.05,1.96)	5.47(4.55,6.60)	0.55(0.42,0.67)
6 months-1 year	159	84	75	346(346,423)	1.68(1.28,2.19)	5.68(4.52,6.90)	0.62(0.48,0.77)
1–2 years	216	101	115	292(246,352)	2.13(1.54,2.86)	5.21(4.24,6.63)	0.56(0.46,0.68)
2–6 years	419	211	208	295(250,346)	2.71(2.13,3.80)	3.41(2.79,4.32)	0.48(0.39,0.60)
6–13 years	860	442	418	277(237,323)	3.15(2.51,4.05)	2.62(2.19,3.19)	0.44(0.37,0.54)
13–18 years	206	101	105	241(203,284)	3.19(2.62,4.00)	2.23(1.89,2.71)	0.41(0.34,0.50)

Footnote: Data are presented as median (25th percentile, 75th percentile)

### Normality test and transformation

Normality of the peripheral blood indices (SII, NLR, PLR, and LMR) was assessed using the Kolmogorov-Smirnov test. The results indicated that none of the parameters followed a normal distribution (*P* < 0.05). Subsequently, the data were transformed using the Box-Cox method. Upon re-evaluation, the transformed data showed no significant deviation from normality (*P* > 0.05) (Table 2).

**Table 2:** Normality test results for SII, NLR, PLR, and LMR before and after transformation

Parameter	Before Transformation			After Transformation		
	Skewness	Kurtosis	K-S Asymptotic Significance	Skewness	Kurtosis	K-S Asymptotic Significance
SII	0.023	-0.275	<0.001	0.000	-0.028	0.972
NLR	0.006	-0.310	<0.001	0.000	-0.029	0.950
PLR	0.040	-0.278	<0.001	0.000	-0.028	1.000
LMR	0.035	-0.051	<0.001	0.016	-0.067	0.192

### Comparison of SII, NLR, PLR, and LMR by sex

**Table 3:** Comparison of SII, NLR, PLR, and LMR levels between males and females

Parameter	T value	*P* value	༺Z-test	
			Z value	Z* value
SII	3.411	0.000	3.411	8.392
NLR	2.838	0.005	2.838	8.392
PLR	2.403	0.016	2.403	8.392
LMR	1.950	0.051	-	-

### Comparison of SII, NLR, PLR, and LMR across age groups

The reference population was stratified by age. One-way ANOVA revealed that the differences in NLR levels between the following adjacent age groups were statistically significant (*P* < 0.05): 6 months-1 year vs. 1–2 years, 1–2 years vs. 2–6 years, and 2–6 years vs. 6–13 years. Similarly, significant differences (*P* < 0.05) were observed for SII, PLR, and LMR between the 1–2 years vs. 2–6 years and 2–6 years vs. 6–13 years age groups. However, subsequent Z-test analysis showed that only for NLR between the 6 months-1 year and 1–2 years groups was the Z-value less than the critical Z*-value, indicating that this specific difference was not clinically significant and did not warrant separate reference intervals. For all other parameters and comparisons where no statistically significant difference was found (*P* > 0.05), partitioning was also deemed unnecessary (Table 4). Based on these results, reference intervals for SII, NLR, PLR, and LMR were established for three consolidated age groups: Group 1 (28 days-2 years, combining the original 28 days-6 months, 6 months-1 year, and 1–2 years subgroups), Group 2 (2–6 years), and Group 3 (6–18 years, combining the original 6–13 years and 13–18 years subgroups).

**Table 4:** Comparison of SII, NLR, PLR, and LMR levels across age groups

Parameter	Age	T value	*P* value	༺-test	
				Z value	Z* value
SII	28 days-6 months	1.676	0.094	-	-
	6 months-1 year	1.102	0.271	-	-
	1–2 years	10.394	< 0.001	10.394	5.520
	2–6 years	10.223	< 0.001	10.223	6.925
	6–13 years	0.960	0.337	-	-
	13–18 years	0.960	0.337	-	-
NLR	28 days-6 months	0.977	0.329	-	-
	6 months-1 year	2.192	0.018	2.192	4.176
	1–2 years	7.710	< 0.001	7.710	5.250
	2–6 years	9.364	< 0.001	9.364	6.925
	6–13 years	0.398	0.691	-	-
	13–18 years	0.398	0.691	-	-
PLR	28 days-6 months	1.654	0.098	-	-
	6 months-1 year	0.199	0.842	-	-
	1–2 years	7.167	< 0.001	7.167	5.250
	2–6 years	8.690	< 0.001	8.690	6.925
	6–13 years	1.258	0.209	-	-
	13–18 years	1.258	0.209	-	-
LMR	28 days-6 months	1.848	0.065	-	-
	6 months-1 year	1.453	0.146	-	-
	1–2 years	5.479	< 0.001	5.479	5.250
	2–6 years	7.719	< 0.001	7.719	6.925
	6–13 years	0.194	0.846	-	-
	13–18 years	0.194	0.846	-	-

### Established reference intervals for SII, NLR, PLR, and LMR

**Fig. 2:**
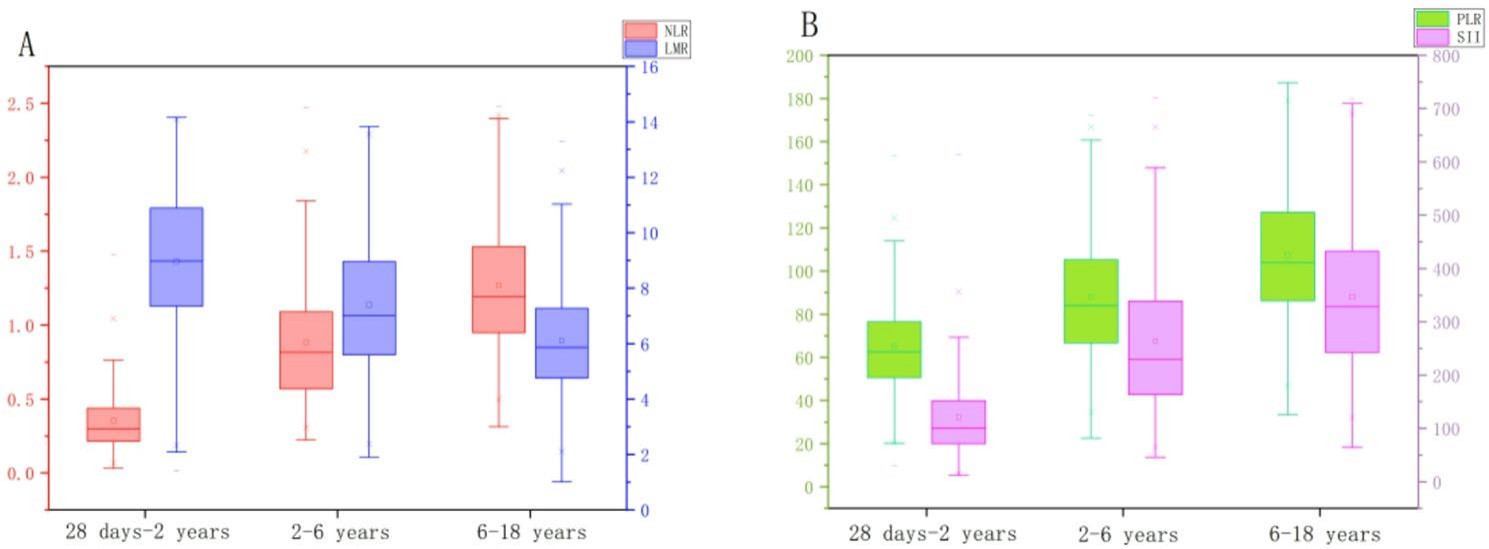
Trends in SII, NLR, PLR, and LMR Across Age Groups, (**A**) Values of NLR and LMR across different age groups; (**B**) Values of PLR and SII across different age groups

Reference intervals for each parameter were established by the nonparametric method for the respective age groups using the percentile method (P₂.₅ to P₉₇.₅) and are presented in Table 5. Analysis of the intervals revealed that SII, NLR, and PLR levels tended to increase with age, whereas LMR demonstrated a decreasing trend (Fig. 2).

**Table 5:** The SII, NLR, PLR, and LMR reference interval calculate by nonparametric methods

Parameter	*n*	Age	Reference intervals
SII(×10⁹/L)	529	28 days-2 years	99.88 (28.60-298.85)
	419	2–6 years	229.71 (83.09-601.33)
	1066	6–18 years	328.71 (140.02-657.19)
NLR	529	28 days-2 years	0.30 (0.10–0.92)
	419	2–6 years	0.81 (0.35–1.88)
	1066	6–18 years	1.19 (0.57–2.30)
PLR	529	28 days-2 years	62.58 (29.39-115.15)
	419	2–6 years	83.98 (39.93-150.46)
	1066	6–18 years	103.79 (56.74-172.55)
LMR	529	28 days-2 years	8.97 (3.42–13.84)
	419	2–6 years	7.00 (2.81–13.03)
	1066	6–18 years	5.86 (2.41–10.56)

### Verification of reference intervals

In accordance with WS/T 402 [18], the established reference intervals were verified using the “healthy population sampling verification method.” A total of 92 children (47 males and 45 females) undergoing health examinations in the Department of Child Health at the First People’s Hospital of Zigong from April to September 2025 were selected for validation. The age distribution was as follows: 31 in the 28 days-2 years group, 29 in the 2–6 years group, and 32 in the 6–18 years group. All validation samples met the inclusion criteriaoutlined in Sect. 1.1 and shared consistent characteristics withthe original cohort used to establish the reference intervals. Following the CLSI EP28-A3C guideline [17], the verification was considered successful if the proportion of validation results falling outside the reference intervals was ≤ 10%. Our results confirmed that the established reference intervals for peripheral blood SII, NLR, PLR, and LMR in children from the Zigong region are suitable and applicable for local clinical practice.

## Discussion

This study is the first to systematically establish age-specific reference intervals for SII, NLR, PLR, and LMR in healthy children aged 28 days to 18 years in the Zigong region of China. Our results indicate no clinically significant sex-based differences for these parameters; however, they exhibited clear age-dependent trends: SII, NLR, and PLR gradually increased with age, while LMR demonstrated a progressive decline. These trends align closely with established patterns of hematopoietic development in children.

During infancy, lymphocytes constitute up to 60% of circulating leukocytes. Neutrophil levels gradually increase during the preschool years, with the neutrophil-to-lymphocyte ratio reaching 1:1 by 4–6 years of age. After age 6, neutrophils become more predominant while lymphocytes decrease, progressively approaching adult proportions [11]. Accordingly, we observed that NLR increased with age, approaching a value of 1 in the 2–6 years group, consistent with this developmental process. PLR also showed a general increasing trend, though it was less pronounced, which can be attributed to the wide reference intervals for platelets throughout childhood [12], even when considering the unique developmental trajectory of lymphocytes [11].

LMR displayed a distinct decline with age, particularly marked between the 28 days-2 years and 2–6 years groups. This sharp decrease corresponds to the period when lymphocytes can reach up to 60% around 2–3 years of age [11], followed by a gradual decline, while monocyte counts remain relatively stable [12]. The decline in LMR was less pronounced from the 2–6 years to the 6–18 years group, consistent with the stabilization of lymphocyte percentages at 20%-40% after approximately 7 years of age [11]. These dynamic changes in SII, NLR, PLR, and LMR are intrinsically linked to the maturation of the pediatric immune system, where ongoing development of immune organs and evolving cellular populations and functions lead to age-associated variations in these inflammatory indices [19]– [20]. These physiological changes underscore the necessity of establishing age-specific reference intervals for children, as the direct application of adult ranges may lead to clinical misinterpretation.

To further evaluate the characteristics of the reference intervals established in this study, we compared them with those reported for children in other regions of China [13–15, 21](Table 6). The results revealed notable differences in the reference intervals for SII, NLR, PLR, and LMR among children of the same age groups across these regions in china. For children aged 28 days to 2 years, Guangdong reported reference intervals of 187.4 (109.8, 219.9) for SII, 0.58 (0.33, 0.68) for NLR, and 69.9 (48.7, 78.5) for PLR. In the 2–6 years age group, Shenyang reported SII, NLR, and PLR values of 179.86 (101.27, 210.95), 0.55 (0.35, 0.83), and 72.58 (59.10, 82.7), respectively. For the same age group, Hebei reported mean SII and NLR values of 640.43 ± 105.43 and 1.05 ± 0.51, respectively (PLR and LMR not provided). For adolescents aged 6–18 years, Shenzhen established reference intervals of 325.75 (231.51, 437.59) for SII, 1.07 (0.73, 1.34) for NLR, 109.32 (86.02, 126.19) for PLR, and 6.67 (5.26, 8.33) for LMR.

**Table 6:** Comparison of reference intervals across different regions in China

Region	Age	*n*	Reference Intervals			
			SII(×10^9^/L )	NLR	PLR	LMR
Guangdong ^13^	28 days-2 years	198	187.4 (109.8,219.9)	0.58 (0.33,0.68)	69.9 (48.7,78.5)	-
Shenyang ^14^	2–6 years	60	179.86 (101.27,210.95)	0.55 (0.35,0.83)	72.58 (59.10,82.7)	-
Hebei ^21^	2–6 years	50	640. 43 ± 105. 43	1. 05 ± 0. 51	-	-
Shenzhen^15^	6–18 years	150	325.75 (231.51,437.59)	1.07 (0.73,1.34)	109.32 (86.02,126.19)	6.67 (5.26,8.33)

Footnote: Data presented are derived from the healthy control groups or reference populations reported in the cited regional studies

Comparisons with studies from other regions in China [13–15, 21]revealed distinct differences in the established reference intervals. Specifically, the SII intervals in our study had lower lower limits and higher upper limits than those reported for corresponding age groups in Guangdong, Shenyang, and Shenzhen, whereas the upper limit for SII in Hebei was 130 units higher than our value. Similarly, our reference intervals for NLR, PLR, and LMR generally demonstrated lower lower limits and higher upper limits compared to other regions. Several factors may explain these regional variations. First, differences in sample size may contribute [20]. Our study featured a larger total sample size (*n* = 2014) with each age subgroup containing more than 120 individuals, adhering to CLSI recommendations [17], which likely yields more robust estimates compared to the smaller samples in other studies [13–15, 21]. Second, regional environmental factors-such as climate, humidity, air quality, and sunlight exposure-may influence respiratory mucosal barrier integrity and immune cell activity, potentially causing fluctuations in these inflammatory markers [22]. Genetic polymorphisms across different populations could also affect the efficiency of immune cell differentiation and maturation, leading to inherent differences in immunological parameters [23]. Furthermore, regional variations in dietary patterns and hygiene practices [24]– [25] may modulate immune system homeostasis, thereby influencing the established reference intervals. These comparative findings reinforce the necessity of establishing region-specific reference intervals for laboratory parameters.

This study was conducted within the specific context of Zigong. While this provides a rigorously controlled cohort for establishing age-specific intervals, the population is not presented as nationally representative. Instead, this work offers a foundational model and a high-quality regional dataset. The observed differences from other Chinese regions highlight the importance of such local initiatives as precursors to larger, multi-center studies aimed at defining national pediatric reference standards.

The establishment of common reference intervals for both sexes was a deliberate decision informed by statistical analysis and clinical guidelines. Although our results indicated statistically significant differences in SII, NLR, and PLR levels between males and females (*P* < 0.05), the critical assessment for reference interval partitioning, as per the CLSI EP28-A3c guideline [17], relies on the comparison of the Z-value to a critical threshold (Z). For all these parameters, the calculated Z-values were markedly lower than the required Z-value of 8.392. This indicates that the observed sex-associated differences, while statistically significant in our large sample, do not reach a magnitude considered clinically relevant for necessitating separate reference intervals. Consequently, the use of unified intervals is both statistically sound and pragmatically advantageous. It simplifies the clinical interpretation and application of these inflammatory indices for healthcare providers, which is a recognized consideration in reference interval design [17], aligning with the goal of providing efficient and robust tools for initial inflammatory assessment in the general pediatric population.

The establishment of age-specific reference intervals for SII, NLR, PLR, and LMR in our healthy Zigong pediatric cohort fulfills a fundamental prerequisite for their rational clinical application. It is crucial to emphasize that these intervals define the expected distribution of these inflammatory indices in a state of health; a value outside these limits does not, in itself, diagnose any specific disease. Instead, their primary utility lies in providing an objective, population-specific baseline for interpretation, transforming routine complete blood count parameters into nuanced tools for clinical assessment, reflecting their growing role as integrative pediatric biomarkers [26].

In clinical practice, these composite indices serve as accessible, integrative markers of systemic inflammatory status. Our data enable their practical translation in several key scenarios relevant to pediatric care: First, for risk stratification and prognostication in children already under clinical management. For a child diagnosed with an infection, Kawasaki disease, or a malignancy, the degree of deviation of their SII or NLR from our established healthy ranges can offer quantitative insight into the severity of the inflammatory response. This can help predict complications - such as intravenous immunoglobulin resistance in Kawasaki disease [2, 27]or worse outcomes in pediatric sepsis [28] - and monitor the trajectory in response to therapy [6, 7]Similarly, in pediatric oncology, pretreatment PLR and NLR have shown prognostic significance for various solid tumors [29]. Without a robust local reference standard, interpreting the magnitude of such deviation remains subjective. Second, in the context of screening and initial assessment of a child presenting with non-specific symptoms (e.g., prolonged fever, fatigue). Markedly elevated SII, NLR, or PLR values, when contrasted against our reference intervals, can act as an objective “red flag.” This prompts the clinician to initiate investigations for underlying disorders, such as differentiating complicated appendicitis [1, 30]or identifying early systemic inflammation [31], thereby guiding targeted diagnostic workup. Third, these intervals are vital for longitudinal monitoring of children with chronic inflammatory conditions. For instance, the lymphocyte-to-monocyte ratio (LMR) and PLR have been identified as useful markers for tracking disease activity in pediatric Crohn’s disease [32]and juvenile idiopathic arthritis [33], respectively. Our reference intervals provide the healthy standard against which such inflammatory burden can be measured over time. Therefore, the clinical value of this work is not in diagnosing disease from an isolated abnormal value, but in equipping practitioners with an essential interpretive framework. By providing locally derived, age-stratified reference intervals, we facilitate more informed clinical suspicion, improve assessment accuracy, and support monitoring within the broader pediatric healthcare strategy for the Zigong region and similar populations.

The interpretability of our findings should be considered within the context of several methodological boundaries. First, the operational definition of a ‘healthy’ pediatric population in this cross-sectional study is inherently bounded by the sensitivity of our screening tools. Our protocol, combining questionnaires and routine blood tests, represents a pragmatic approach for reference interval establishment, yet it may not capture very early or subclinical inflammatory states identifiable only through longitudinal follow-up or specialized assays. This reflects a fundamental trade-off between feasibility and diagnostic comprehensiveness in large-scale reference value studies. Second, a key limitation, and one directly pertinent to the reviewer’s inquiry, is the geographic specificity of our cohort, which was recruited solely from the Zigong region. The selection of Zigong was based on pragmatic access to a well-characterized pediatric healthcare system, which ensured rigorous internal consistency in recruitment and measurement protocols - a critical strength for establishing a high-quality foundational dataset. This study provides the first systematic reference intervals for these novel inflammatory indices in this region, thereby addressing a local data gap and offering a vital calibration tool for clinical laboratories in Zigong. We explicitly acknowledge that this single-center design does not purport our population to be nationally representative. The reported intervals reflect the integrated physiological and environmental milieu of children in this specific locale. Consequently, their direct applicability to other Chinese populations with differing genetic backgrounds, diets, or environmental exposures requires explicit verification. This work should thus be viewed as a necessary and rigorous regional benchmark, forming a foundational dataset for future multi-center studies aimed at defining national pediatric standards. Third, as our reference population was recruited from children presenting for routine health checkups, it may reflect families with greater health awareness or access to healthcare, introducing potential selection bias and limiting the generalizability of the intervals to all socioeconomic groups within the region. Other unmeasured confounders, such as detailed dietary habits, recent minor infections not reported in questionnaires, or genetic background variations, could also influence the inflammatory indices. Therefore, these intervals are primarily recommended for use as a local clinical calibration tool, with their external validity to other populations awaiting future confirmation.

## Conclusion

In conclusion, this study establishes reference intervals for SII, NLR, PLR, and LMR in children from the Zigong region, thereby providing a foundational tool for local pediatric practice. The primary contribution of our work is not to enable direct diagnosis from a single value, but to equip clinicians with a robust, context-aware interpretive framework. This framework transforms routine blood count ratios into valuable tools for raising suspicion, aiding assessment, and monitoring disease progression within the broader clinical decision-making process. Future multicenter studies are warranted to validate these intervals and further explore the diagnostic and prognostic utility of these indices in specific childhood diseases.

## Data Availability

The data supporting the findings of this study are available from the corresponding author upon reasonable request.
